# Evaluation of Albumin, Transferrin and Transthyretin in Inflammatory Bowel Disease Patients as Disease Activity and Nutritional Status Biomarkers

**DOI:** 10.3390/nu15153479

**Published:** 2023-08-07

**Authors:** Małgorzata Godala, Ewelina Gaszyńska, Konrad Walczak, Ewa Małecka-Wojciesko

**Affiliations:** 1 Department of Nutrition and Epidemiology, Medical University of Lodz, 90-752 Lodz, Poland; ewelina.gaszynska@umed.lodz.pl; 2 Department of Internal Medicine and Nephrodiabetology, Medical University of Lodz, 90-419 Lodz, Poland; konrad.walczak@umed.lodz.pl; 3 Department of Digestive Tract Diseases, Medical University of Lodz, 90-647 Lodz, Poland; ewa.malecka-panas@umed.lodz.pl

**Keywords:** Crohn’s disease, ulcerative colitis, malnutrition, albumin, transthyretin, transferrin, nutritional status disease activity

## Abstract

Inflammatory bowel disease (IBD), which includes Crohn’s disease (CD) and ulcerative colitis (UC), is often accompanied by malnutrition that manifests itself as nutrient deficiencies and body mass loss or deficit. The purpose of this study is to evaluate the utility of albumin, transferrin and transthyretin levels in the assessment of nutritional status and IBD activity. The case–control study included 82 IBD patients. The serum concentrations of albumin, transferrin and transthyretine were determined by a quantitative sandwich enzyme-linked immunosorbent assay (ELISA). Significantly lower median concentrations of albumin were found in the IBD patients vs. controls and in CD patients compared to the UC patients. Significantly higher median transthyretin concentrations were found in the IBD patients compared to the healthy subjects. There were no significant differences in median transferrin concentrations between the IBD patients and the healthy subjects. Significantly higher albumin levels were found in IBD patients in remission compared to patients with moderate and severe exacerbation of IBD symptoms. There were no significant differences in the median transferrin or transthyretin levels in patients with IBD depending on disease activity. No differences were identified in the median transferrin or transthyretin levels in the IBD patients according to nutritional status. The median albumin concentrations in the IBD subjects were significantly higher in patients with normal body fat, normal BMI and normal waist circumferences compared to those with an abnormal nutritional status. The albumin levels reflect both nutritional status and disease activity and therefore cannot be considered a prognostic marker of malnutrition in IBD. As regards the utility of transferrin and transthyretin as markers of activity and nutritional status in IBD patients, further studies are required.

## 1. Introduction

The European Society for Clinical Nutrition and Metabolism (ESPEN) defines malnutrition as “a state of abnormal body composition resulting from lack of intake or uptake of nutrition that leads to altered body composition (decreased fat free mass) and body cell mass leading to diminished physical and mental function and impaired clinical outcome from disease” [1]. Inflammatory bowel disease (IBD), which includes Crohn’s disease (CD) and ulcerative colitis (UC), is often accompanied by malnutrition that manifests itself as nutrient deficiencies and body mass loss or deficit [2,3,4]. The prevalence of malnutrition in IBD patients is 20–85% [3,4,5,6,7,8,9,10,11,12,13,14]. In particular, malnutrition accompanies the acute phase of CD, as a consequence of disturbed gastrointestinal function, impaired nutrient absorption and immune function, which further exacerbates the inflammatory process. About 20% of the patients suffer from kwashiorkor malnutrition, which is one form of protein–energy malnutrition requiring parenteral nutrition [15]. There are also emerging data indicating the prevalence of obesity in these patients, which is probably related to the achievement of sustained remission [16,17].

The causes of malnutrition include an inadequate dietary energy supply, which results from a reduction in food intake due to poor appetite or the elimination diets introduced by patients themselves with no justified reasons [5,6,7,8,9]. Another cause is the accompanying malabsorption syndrome resulting from the loss of intestinal villi due to inflammation, the presence of fistulas or small bowel resection. Another quite frequent problem is overgrowth of the small intestinal bacterial flora due to, among others, impaired passage through inflamed Bauhin’s valve stenosis [6,10,11]. Drug interactions with food components also have a significant impact on the onset of malnutrition. Sulfasalazine, the first-line drug used in IBD, blocks folic acid absorption, whereas steroids impair intestinal calcium resorption [12,13]. Another cause of nutritional abnormalities is an excessive loss of nutrients with diarrhea, as well as through inflammation-damaged intestinal mucosa. Protein loss through this mechanism is very high, particularly in CD, causing hypoalbuminemia in most patients with the disease recurrence [14,15]. In IBD exacerbation, the demand for energy, essential nutrients and substances increases, which is not compensated for by the adequate increase in the supply of energy and nutrients from the diet. This leads to progressive malnutrition, which affects not only patients with low body weight but also those with a normal nutritional status assessed based on anthropometric indices. Hence, assessing the nutritional status of patients with IBD with anthropometric measures (BMI, waist circumference and body composition) may be insufficient, and early nutritional status abnormalities may not be identified. 

Therefore, we aimed to evaluate the serum concentrations of albumin, transferrin and transthyretin as biochemical markers of the nutritional status in patients with IBD [18,19,20,21,22,23]. 

Albumin is a marker of malnutrition widely discussed in the literature. It is a protein produced by hepatocytes and present in the serum at a concentration of 35–50 g/L. Its production depends on the supply of amino acids, plasma oncotic pressure, the concentration of cytokines (mainly IL-6) and the number of active hepatocytes [18]. Albumin is a factor regulating the volume of the intravascular space. Albumin also belongs to the nonspecific transporters of hormones, vitamins, calcium, magnesium, bilirubin, fatty acids and lipids, as well as drugs. The main cause of hypoproteinemia is a decreased albumin concentration, which can result from a high loss of plasma proteins, as in inflammatory conditions of the intestinal and gastric mucosa such as CD and UC [20,21]. A meta-analysis of 111 studies conducted in 2017 on a group of nearly 53,000 patients evaluating 43 blood markers of malnutrition identified albumin as a reliable marker of the nutritional status, with the strongest correlation with the results of questionnaires, such as the Nutritional Risk Screening 2002 (NRS-2002), Mini Nutritional Assessment (MNA), Malnutrition Universal Screening Tool (MUST) and Subjective Global Assessment (SGA). However, the authors of this meta-analysis suggested that the determination of albumin alone may lead to an underestimation of the cases of minor malnutrition when the albumin concentration is still normal and may not be recognized by anthropometric and questionnaire assessments. Therefore, they emphasized that other proteins, such as prealbumin (transthyretin), should also be measured [24]. 

Transthyretin is a thyroxine-binding protein, mainly synthesized in the liver. Among the proteins analyzed, it has the shortest half-life (about two days) and is considered a better indicator of the early deficiencies of visceral proteins that may not be detected by the albumin or transferrin concentration alone [21,22]. In the abovementioned meta-analysis, the authors suggested that the transthyretin concentration is a more reliable tool for assessing early malnutrition states not recognized by anthropometric or questionnaire methods than the albumin level [24]. 

Transferrin is used in nutritional assessments as a marker reflecting the progression and severity of the disease [24,25,26,27,28,29]. Its concentration is related to the iron metabolism, while a decrease in its level and escape from the circulation are the result of cytokine responses to metabolic stress [30]. The serum transferrin concentration is less dependent on the energy value of a diet and more dependent on the protein supply [19]. Due to its short half-life, transferrin, similar to transthyretin, is considered a good indicator used for monitoring rapidly occurring changes in the composition of visceral proteins and the effectiveness of nutritional therapy monitoring [18].

The search for new simple indicators of IBD activity and the nutritional status is ongoing. It is essential for planning the management, monitoring and treatment of those diseases [4,8,10,14,15].

The purpose of this study is to evaluate the utility of the albumin, transferrin and transthyretin levels in the assessment of the nutritional status and IBD activity.

## 2. Material and Methods

### 2.1. Study and Control Groups 

The case–control study included 82 IBD patients, 42 women (51.2%) and 40 men (48.8%) (mean age 38.1 ± 11.6 years); 48 patients with CD and 34 patients with UC, treated at the Department of Digestive Tract Diseases, Medical University of Lodz. All the subjects had been diagnosed with IBD based on clinical presentation and endoscopic and histopathological examinations. Individuals suffering from conditions that could adversely affect their nutritional status, such as kidney disease, cancer and cardiovascular failure, were excluded from the study. The control group consisted of 25 healthy volunteers (mean age 38.6 ± 9.1 years).

The study was approved by the Bioethics Committee of the Medical University of Lodz (No. RNN/70/22/KE). All the subjects gave written consent to participate in the study. 

### 2.2. Disease Activity

In the case of CD patients, the Crohn’s Disease Activity Index (CDAI) and the Montreal classification were applied. According to the guidelines of the European Crohn’s and Colitis Organization (ECCO), CDAI < 150 was defined as disease remission, CDAI 150–220 as a mild exacerbation of symptoms and CDAI 220–450 as a moderate exacerbation of symptoms, while CDAI > 450 was considered a severe exacerbation of disease symptoms. The Montreal classification specifies age at diagnosis (<16, 17–40 or >40); location (ileal, colonic or ileocolonic) and behavior (non-stricturing/non-penetrating, stricturing or penetrating) [31].

In the case of UC patients, the Partial Mayo Score and the Montreal classification were applied. A Partial Mayo Score of 0 corresponded to clinical remission, while mild activity of the disease was diagnosed for a Partial Mayo Score of 1, moderate for a Partial Mayo Score of 2 and severe for a Partial Mayo Score of 3 [29]. According to the Montreal classification based on the extent of lesions identified in the large intestine, the following distinctions were made: ulcerative proctitis (E1)—mucosal lesions limited to the rectum, left-sided colitis (E2)—involvement limited to the portion of the colorectum distal to the splenic flexure and pancolitis (E3)—involvement extents proximal to the splenic flexure or lesions affecting the entire large intestine. Based on the severity of the exacerbations (flare-ups), the following forms of clinical remission were distinguished: an asymptomatic form (S0); mild exacerbation (S1), symptoms: up to four stools per day (with or without blood), no general symptoms, ESR normal; moderate exacerbation (S2), symptoms: more than four stools per day, moderate general symptoms; severe exacerbation (S3), symptoms: more than six black stools per day, HR > 90/min, temperature ≥ 37.5 °C, Hb < 10.5 g%, ESR ≥ 30 mm/h [32].

### 2.3. Nutritional Status

The waist circumference was measured in all the subjects, and the BMI (body mass index) was determined by dividing the body weight expressed in kilograms by height in square meters. Weight changes during the last 6 months were reported by the patients. Weight loss more than 5% of the normal patients’ weight was considered important. The body composition of each study participant was assessed by a bioelectrical impedance analysis (BIA) using InBody 270. Fat, water, muscle mass and lean body mass were measured. 

### 2.4. Blood Sample Collection and Serum Markers of Nutritional Status

Fasting blood samples for the laboratory tests were collected from the ulnar vein and then centrifuged (2000× *g* for 20 min). The isolated serum was frozen at −80 °C. The obtained samples were used to determine the inflammatory markers.

The serum concentrations of albumin, transferrin and transthyretine were determined by a quantitative sandwich enzyme-linked immunosorbent assay (ELISA) using kits from Biorbyt LLC, Durham, NC, USA. All the tests were performed according to the manufacturer’s instructions. Additionally, laboratory tests, such as an automated complete blood count, were also performed. 

### 2.5. Statistical Analysis

All the computational procedures were performed using Statistica™ 14 (TIBCO Software Inc., Palo Alto, CA, USA). Categorical variables were described with the use of integers and percentages. Numerical traits were depicted by means, standard deviations, medians and lower-to-upper quartiles. The normality of distribution was preliminarily assessed using the Shapiro–Wilk W test. For univariate analyses, the Mann–Whitney *U* test was adjusted when a grouping variable was dichotomous, or the Kruskal–Wallis H test was carried out when a grouping variable had more than two categories. Correlations were assessed by the Spearman’s coefficient (r). For multivariate procedures, generalized linear models (for non-normally distributed variables) were performed in order to appraise differences in numerical variables between the study groups. All the multivariate models were controlled for age, gender, educational level, family history and disease duration of the study participants. A level of *p* < 0.05 was deemed statistically significant. 

## 3. Results

### 3.1. Characteristics of the Study Subjects

The study and control groups were similar in terms of age and gender. There were no differences between the two groups in terms of the prevalence of smoking or the mean values of the BMI and waist circumferences. In contrast, significant differences were found in the body compositions of the subjects. The patients with IBD had significantly lower mean body fat compared to the control group. The mean duration of the disease was 8.4 ± 5.7 years. One in four received biological treatment (*n* = 66, 80.5%) and took oral preparations of 5-aminosalicylic acid (*n* = 64, 78.0%). One in three subjects used immunosuppressants (*n* = 33, 40.2%) and corticosteroids (*n* = 25, 30.5%) (Table 1). 

Among the IBD patients, nearly half of the subjects (*n* = 40, 48.8%) were in clinical remission without any disease symptoms, including 23 patients (47.9%) with CD and 17 with UC (50%). Endoscopic remission was confirmed in 17 patients with CD and in 17 patients with UC (Table 2).

### 3.2. Concentrations of Albumin, Transferrin and Transthyretin in CD and UC Patients

Significantly lower median concentrations of albumin were found in the CD patients compared to the UC patients (15.75 mg/mL vs. 32.70 mg/mL, *p* < 0.0001). The median albumin concentrations in the CD and UC patients were also significantly lower than in the healthy subjects (43.20 mg/mL, *p* < 0.0001).

There were no significant differences in the median transferrin concentrations between the IBD patients and the healthy subjects. The individuals with CD and UC had slightly lower median concentrations of transferrin compared to the controls; however, this difference was not statistically significant (1.32 mg/mL vs. 1.55 mg/mL vs. 1.90 mg/mL, *p* = 0.3548, respectively).

Significantly higher median transthyretin concentrations were found in the CD patients compared to the healthy subjects (3.02 ng/mL vs. 1.4 ng/mL, *p* < 0.0001) and in the UC patients compared to the healthy subjects (2.82 ng/mL vs. 1.40 ng/mL, *p* < 0.0001). There were no statistically significant differences in the median transthyretin levels between the CD and UC patients (Table 3).

In the IBD patients, there were no differences in the albumin or transferrin concentrations in terms of age, gender, place of residence, educational level, smoking or alcohol consumption. In contrast, significantly lower transferrin levels were observed in patients with a positive family history of IBD compared to those with a negative family history (1.1 mg/mL vs. 1.7 mg/mL, *p* < 0.05). This relationship was not confirmed for albumin. The transthyretin concentration did not differ according to gender, place of residence, educational level, positive family history or smoking in the IBD patients. In contrast, significantly higher median transthyretin levels, as compared to the other subjects, were found among patients aged under 40 (3.2 ng/mL vs. 2.6 ng/mL, *p* < 0.01). 

### 3.3. Concentrations of Albumin, Transferrin and Transthyretin vs. Disease Activity 

No significant differences were found in the median albumin levels in the IBD patients according to disease duration, use of biological treatment or history of surgical treatment of the disease. 

No significant differences were identified in the transferrin concentrations in the IBD patients according to disease duration, use of biological treatment or history of surgical treatment of the disease.

There were no significant differences in the median transthyretin concentrations in the IBD patients according to disease duration or history of surgical treatment of the disease. In contrast, significantly higher median levels of transthyretin were found in patients on biologic therapy compared to the remaining IBD patients (3.1 ng/mL vs. 2.1 ng/mL, *p* < 0.05) (Table 4).

There were no significant differences in the transferrin or transthyretin concentrations in the UC patients depending on disease activity, as assessed by the Montreal classification. On the other hand, significantly higher albumin levels were found in patients in remission compared to patients with moderate and severe exacerbation of UC symptoms (Partial Mayo Score of 0 vs. 2 vs. 3—42.5 mg/mL vs. 28.5 mg/mL vs. 17.6 mg/mL, respectively, *p* = 0.0151) (Table 5). 

In the CD patients, higher median concentrations of albumin were found in patients in remission (CDAI < 150) as compared to those with mild, moderate and severe symptoms (47.2 mg/mL vs. 19.7 mg/mL vs. 17.2 mg/mL vs. 13.7 mg/mL, respectively, *p* = 0.0334). Additionally, a significantly higher median concentration of albumin was observed in patients diagnosed after the age of 40 than in those diagnosed at an earlier age (A3 vs. A2 vs. A1—23.6 mg/mL vs. 17.2 mg/mL vs. 9.4 mg/mL, respectively, *p* = 0.0488). There were no significant differences in the median transferrin or transthyretin levels in patients with CD depending on disease activity, as assessed by the Montreal scale and CDAI (Table 6).

### 3.4. Concentrations of Albumin, Transferrin and Transthyretin and the Nutritional Status of Patients

No differences were identified in the median transferrin or transthyretin levels in the IBD patients according to the BMI, waist circumference, unintentional weight loss in the preceding six months or body composition. In contrast, significantly higher median concentrations of albumin were observed among patients with a normal BMI compared to those with a low BMI (<18.5kg/m^2^) (34.3 mg/mL vs. 28.1 mg/mL, respectively, *p* = 0.0021). Also, the median albumin concentration in patients with a normal BMI was significantly higher than in those with high BMI values indicative of overweight and obesity (34.3 mg/mL vs. 18.4 mg/mL vs. 14.8 mg/mL, respectively, *p* = 0.0251). Furthermore, significantly higher median levels of albumin were found in patients with a normal waist circumference compared to those with visceral obesity (27.8 mg/mL vs. 16.8 mg/mL, respectively, *p* = 0.0213). In addition, higher median albumin levels were observed in patients reporting a significant weight reduction in the preceding six months than in those with a weight reduction of less than 10% compared to the baseline body weight (27.1 mg/mL vs. 20.6 mg/mL, respectively, *p* = 0.0463). The median albumin concentrations in the IBD subjects were significantly higher in patients with normal body fat compared to those with too-low and too-high body fat (34.1 mg/mL vs. 22.6 mg/mL vs. 13.7 mg/mL, respectively, *p* = 0.04866) (Table 7). These relationships were not confirmed in the group of healthy subjects in whom albumin concentrations did not differ significantly depending on the BMI, waist circumference or body fat content.

### 3.5. Correlations between Albumin, Transferrin and Transthyretin

**Figure 1:**
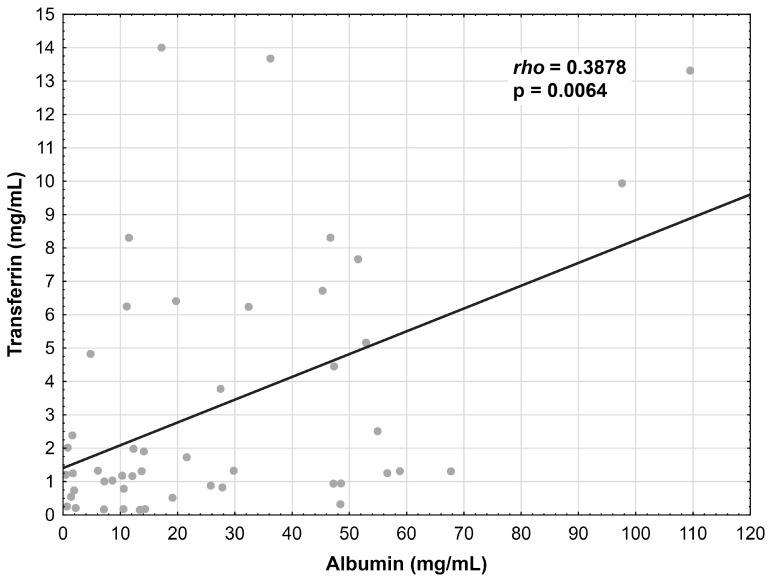
Correlation between the albumin and transferrin concentrations in patients with CD.

**Figure 2:**
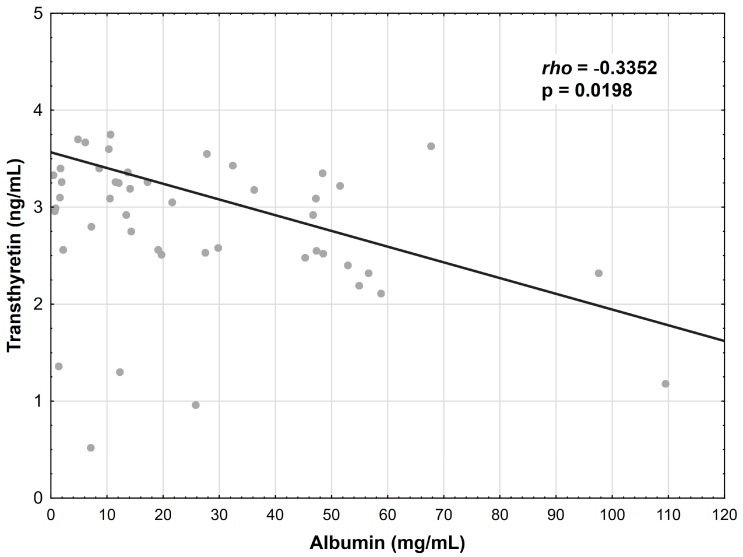
Correlation between the albumin and transthyretin concentrations in patients with CD.

**Figure 3:**
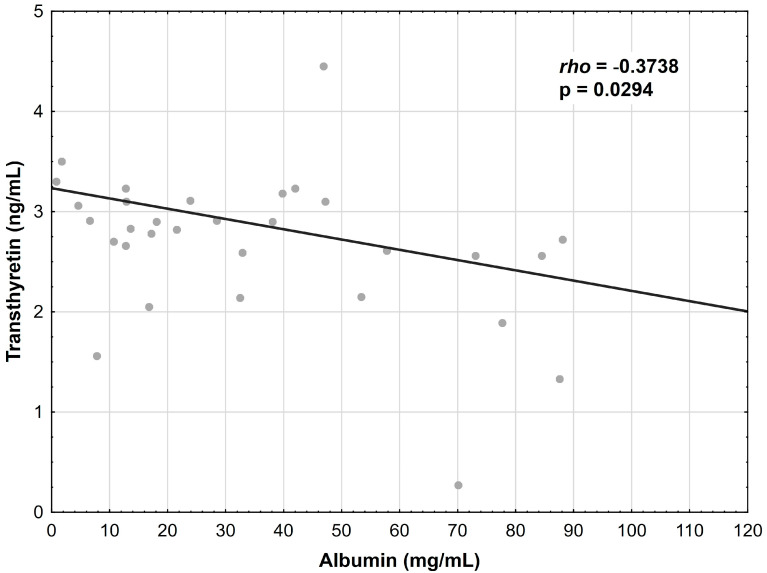
Correlation between the albumin and transthyretin concentrations in patients with UC.

The albumin concentration showed a positive correlation with the transferrin levels in the CD patients (r = 0.3878, *p* = 0.0064) (Figure 1); however, this relationship was not confirmed in the UC group. Additionally, the albumin concentration correlated negatively with the transthyretin concentration in the CD patients (r = −0.3352, *p* = 0.0198) (Figure 2) and in the UC patients (r = −0.3738, *p* = 0.0294) (Figure 3).

## 4. Discussion

In our study, we showed significantly lower albumin levels in the patients with CD and UC compared to the healthy subjects. Our results are consistent with those reported in other studies. 

A study by Wang et al., conducted on a group of 179 patients with IBD, assessed the relationship between AGR (albumin-to-globulin ratio) and IBD. It showed significantly lower concentrations of albumin in patients with CD and UC compared to healthy subjects [33].

Similarly, Mijač et al. demonstrated significantly lower concentrations of albumin in IBD patients than in healthy subjects. The authors of this study concluded that the albumin concentration, along with BMI, were the best predictors of malnutrition in IBD patients [34].

Similar data were also obtained in a study by Geerling et al., in which significantly lower albumin levels compared to healthy subjects were characteristic of both CD and UC patients [35].

Some papers have reported no significant differences in albumin concentrations between patients with IBD and healthy patients. In a study by Capristo et al. conducted among patients with CD, there were no significant differences in the albumin levels between diseased individuals and healthy controls [36]. 

In a study by Lanfranchi et al., the albumin levels in IBD patients were not significantly different from those found in healthy subjects. However, it should be emphasized that, in this study, all IBD patients were in remission, which may explain the discrepancy with the results of our study [37].

In our study, we identified significant differences in the albumin levels depending on the disease activity. Higher albumin concentrations were observed in the CD patients who were in remission than in those with mild, moderate and severe exacerbation of their symptoms. Higher albumin levels were also found in patients with UC in remission compared to individuals with moderate and severe exacerbation of their symptoms.

The study by Wang et al. evaluated the albumin levels in relation to IBD activity. They used the Harvey Bradshaw Index (HBI) score for CD patients and the Mayo score for UC patients. Lower albumin concentrations were noted in patients with a higher severity of symptoms [33].

In a study by Banjamin et al., significantly higher albumin concentrations were found in patients in remission compared to those with active IBD [38]. 

Similarly, a study by Vagianos et al. showed higher serum levels of albumin in patients in remission compared to those with an exacerbation of IBD symptoms [39].

In our study, higher albumin levels were recorded in the patients diagnosed after the age of 40 compared to younger age groups. This confirms observations concerning a more severe course of the disease among patients diagnosed at an earlier age [40,41,42].

A study by Loly et al. including 361 patients with CD showed that a diagnosis at an earlier age (before the age of 40) was associated with a greater severity of symptoms and a higher incidence of IBD strictures [40]. 

A study by Seksik et al. conducted among CD patients found that a diagnosis made before the age of 40 was associated with a higher risk of severe disease activity [42].

In the present study, no significant differences were found in the transferrin levels between the IBD patients and the healthy subjects. Additionally, we did not observe differences in the transferrin concentrations depending on IBD activity.

On the other hand, Matusiewicz et al. showed significantly lower transferrin concentrations in patients with active CD and UC compared to healthy subjects. Also, a positive correlation was found between the transferrin concentrations and indicators of anemia, especially the iron levels [43]. 

Alves et al. showed significantly lower transferrin concentrations in patients with active UC compared to remission; however, they did not find this correlation for CD [44].

Transferrin is considered a negative acute phase reactant, and therefore, its concentration decreases in response to inflammation, as confirmed in studies [43,45,46]. Moreover, by iron sequestration, transferrin prevents its involvement in the production of oxygen-free radicals in the Haber–Weiss reaction, constituting an important element of the serum antioxidant defense. The lack of differences in the transferrin levels between the IBD patients and the controls in our study may result from the anti-TNF therapy administered, which probably improved the iron levels and availability, thereby increasing the transferrin levels [47,48]. Such a relationship was demonstrated by Repnik et al. [48], whose study confirmed an increase in transferrin levels in patients with CD after the implementation of adalimumab therapy. Certainly, the validity of transferrin determination as a prognostic factor in IBD needs to be reevaluated in a larger population and with the use of various pharmacotherapeutic methods. 

In the present study, the transthyretin levels were significantly higher in the group of IBD patients than in the healthy controls. 

Different data were obtained in a study by Koofy et al., who found that the transthyretin levels were insignificantly lower in patients with CD than in healthy subjects [49].

A study by Roma et al. evaluated the albumin levels in a group of pediatric patients with CD. They found no significant differences in the transthyretin levels between the groups of patients and healthy controls [50].

In our study, there were no significant differences in the transthyretin levels in the UC patients depending on disease activity, as assessed by the Montreal classification and the Partial Mayo Score, or in the CD patients depending on disease activity, as assessed with the use of the Montreal and CDAI scales.

In a study by Piras et al. including 44 patients with CD, reduced transthyretin levels were found in patients during exacerbation of their symptoms [51]. 

Similar data were obtained in a study by Weeke et al., in which significantly lower transthyretin concentrations were determined in patients with active CD [52].

Similarly, reduced transthyretin levels were determined in patients with rheumatoid arthritis during exacerbation of their symptoms [53].

Determination of the transthyretin level as a potential marker of the nutritional status and IBD activity requires further study. Its concentration depends on a number of factors, such as the use of corticosteroid therapy, high doses of nonsteroidal anti-inflammatory drugs or treatment with infliximab, which has been reported to increase transthyretin concentrations in patients with IBD [54]. In our study, the majority of patients received biological treatment, which may explain the discrepancies between our results and those obtained in the cited studies. Additionally, in the literature, there are papers that indicate a large contribution of the dietary factor in the regulation of transthyretin concentrations, with particular consideration given to the amount of dietary protein intake [54,55,56].

In a further part of our study, we searched for a relationship between the concentration of visceral proteins and the nutritional status of the patients. We found significantly higher albumin concentrations in the individuals with a normal nutritional status as assessed by the BMI and waist circumference, as well as in the patients with normal body fat. Interestingly, both malnourished and obese (according to the body mass index and body fat content) patients had significantly lower albumin concentrations compared to those with a normal nutritional status.

The relationship between albumin concentration and the BMI has been evaluated in studies conducted on different groups of patients. In the study by Mijač et al., decreased albumin levels were significantly more frequent in malnourished patients compared to those with a normal BMI, thus confirming the findings of the current study [34]. 

A study by Snipelisky et al. showed that lower serum albumin levels in patients with pulmonary arterial hypertension were associated with a higher risk of comorbidities, as well as worse survival and clinical course of the disease. The authors suggested that serum albumin levels can be used as a nonselective marker of the disease severity [57].

A study by Lai et al. conducted on a group of 228 elderly people showed that the concomitant presence of low albumin and BMI in the elderly was associated with a poor prognosis for many diseases [58]. These findings have also been confirmed in other studies [59,60,61,62,63]. It has been shown that low albumin levels and/or low BMI values are associated with higher mortality rates and a worse prognosis in hemodialyzed and oncological patients. Low serum levels of albumin [61,64] and lower BMI values [65] may be associated with chronic inflammation that accompanies many diseases, including IBD [66,67].

In our study, we showed low albumin concentrations not only in the underweight group but also in the obese patients. 

Similar data were obtained in a study by Filliatre-Clement et al., in which low albumin concentrations were presented by both malnourished and obese patients with acute myeloid leukemia (AML). This study evaluated the prognostic value of the BMI and serum albumin levels in these patients, as well as the relationship between the albumin levels and BMI. The authors considered the serum albumin levels to be an independent prognostic factor in AML more reliable than the BMI in terms of the nutritional assessment of patients at the time of diagnosis [68].

In our study, we showed lower albumin levels in patients with a large reduction in body weight occurring over the preceding six months. This might be a reflection of the severity of the IBD damage and the consequences of the food intake and nutritional state of patients with IBD. Additionally, this is probably due to the chronic inflammation that accompanies obesity. In obese individuals, there is a predominance of Th1 lymphocytes over Th2 lymphocytes in the adipose tissue, which leads to the increased secretion of proteins that stimulate macrophages and, thus, induce inflammation [69]. In the case of a significant reduction in body weight and, thus, in the adipose tissue, there is a change in the inflammatory parameters that accompany IBD and are related to the albumin levels [69]. Cells of the inflammatory infiltrate, mainly neutrophils and macrophages, release mediators such as the tumor necrosis factor TNF-α and interleukins (IL-6, IL-1 and IL-8), which leads to a decrease in certain proteins, including albumin. On the other hand, the state of malnutrition is also related to immunological processes and may be induced by the development of inflammation. This occurs due to appetite suppression as a result of an increased release of IL-1 from the inflamed intestine and centrally released 5-hydroxytryptamine [69,70]. Furthermore, nutrients can modulate inflammatory responses in the intestinal ecosystem and act as the components of the cell membrane mediating the expression of the proteins involved in the inflammatory processes, such as cytokines, adhesion molecules and inducible nitric oxide synthase (iNOS) [70]. Not only do cytokines exacerbate the inflammatory process in the intestines but also induce systemic effects in IBD patients, such as hypoproteinemia, by decreasing albumin mRNA expression and reducing albumin synthesis in hepatocytes [71]. Cytokines also affect capillaries by increasing their permeability, which promotes albumin loss [72]. It is difficult to determine unequivocally whether low albumin levels are a consequence of an abnormal nutritional state or inflammation that accompanies IBD. Therefore, determination of the albumin levels in IBD patients appears not to be a particularly promising marker of both the nutritional status and disease activity.

The transferrin and transthyretin concentrations in our study did not differ significantly in the group of IBD patients depending on their nutritional status. In contrast, we found a positive correlation between the transferrin and albumin concentrations in the group of patients, which might suggest the possible relevance of its determination for monitoring IBD. In contrast, the transthyretin concentrations in our study correlated inversely with the albumin concentrations in both the CD and UC patients.

A study by Matusiewicz et al. found a positive relationship between the transferrin and albumin levels, which was more pronounced in the UC patients [43].

Recommendations concerning the use of transthyretin as a marker of the nutritional status have been a topic of debate and controversy for decades. Many scientific societies, including ASPEN and ESPEN, have shared a critical opinion on the relevance of transthyretin determination as a potential marker of the nutritional status [55,56,73]. On the other hand, in countries like China, Korea, Japan, Indonesia and Australia, transthyretin is routinely determined for the assessment of the nutritional status in hemodialyzed, oncological and terminally ill patients [74,75,76,77,78,79]. In light of the cited study results, it could be hypothesized that the transthyretin levels in IBD patients should rather be considered as an early marker of malnutrition, strongly correlated with the dietary protein intake and short-term changes in the patient’s body composition. This hypothesis was not confirmed in our study.

Our study had some limitations. It was conducted on a small group of patients with a relatively good nutritional status assessed with the use of anthropometric methods. Moreover, the vast majority of patients were on biological treatments, which could indirectly affect the markers determined due to the reduction of inflammation. Therefore, caution should be exercised when interpreting the serum protein measurements, especially in patients with infection, acute inflammation or recent trauma. Further research is required to assess the validity of determining the concentrations of albumin, transferrin and transthyretin in patients with IBD.

In the light of our study, the biomarkers assessed—particularly the albumin levels—might be useful in terms of controlling IBD activity. We found significantly higher albumin concentrations in the patients in clinical remission. Additionally, we found lower albumin concentrations in the malnourished patients—both undernourished and overnourished patients. The relationship between the albumin concentration and nutritional status as measured by the BMI, waist circumference and body fat content was not confirmed in the healthy subjects. Therefore, our observations support the impact of inflammation on the reduction of the albumin concentration. In contrast, the transferrin levels were significantly lower in patients with a positive family history of IBD. This encourages the search for a relationship between the transferrin concentrations and a family history of IBD.

## 5. Conclusions

The albumin levels reflect both the nutritional status and disease activity and therefore cannot be considered a prognostic marker of malnutrition in IBD. As regards the utility of transferrin and transthyretin as markers of the activity and nutritional status in IBD patients, further studies are required.

## Figures and Tables

**Table 1:** General characteristics of the study participants.

	IBD N(%)/Mean ± SD	Controls N(%)/Mean ± SD
CD	48 (58.5)	-
UC	34 (41.5)	-
Age (years)	38.1 ± 11.6	38.6 ± 9.1
Female	42 (51.2)	15 (60)
Level of education		
Secondary	42 (51.2)	4 (16) *
High	40 (48.8)	21 (84) *
Current smoking	14 (17.1)	3 (12)
Disease duration	8.4 ± 5.7	-
Surgery history	22 (26.8)	-
Weight lost during last 6 months (%)	50 (60.9)/16.5 ± 8.2	-
Anthropometry		
BMI (kg/m^2^)	24.23 ± 4.76	24.64 ± 3.97
Waist circumference (cm)	88.88 ± 14.54	85.28 ± 9.06
Fatty tissue (%)	27.1 ± 9.6	30.1 ± 8.6 *
Medications		
Biological therapy	66 (80.5)	-
Immunosuppression	33 (40.2)	-
Steroids	25 (30.5)	-
5-ASA	64 (78.0)	-

* *p* < 0.05.

**Table 2:** Disease activity of the study participants.

	CD *n* (%)	UC *n*(%)
Self-reported clinical stage of disease		
Remission	23 (47.9)	17 (50)
Moderate	16 (33.3)	11 (32.4)
Active	15 (31.2)	6 (17.6)
CDAI		
0	17 (35.4)	-
1	10 (20.9)	-
2	17 (35.4)	-
3	4 (8.3)	-
Montreal classification		
Age at diagnosis (A1/A2/A3)	8 (16.7)/36 (75)/4 (8.3)	-
Disease location (L1/L2/L3)	17 (35.4)/7 (14.6)/24 (50)	-
Disease behavior (B1/B2/B3)	21 (43.8)/18 (37.5) /15 (3.2)	-
Partial Mayo Score		
0	-	17 (50.0)
1	-	0 (0)
2	-	11 (32.4)
3	-	6 (17.6)
Montreal classification		
Disease location (E1/E2/E3)	-	4 (11.8)/16 (47.0)/14 (41.2)
Severity of relapse (S0/S1/S2/S3)	-	12 (35.3)/10 (29.4)/11 (32.4)/3 (8.8)

**Table 3:** Concentrations of albumins, transferrin and transthyretin in the study participants.

Analyzed Marker	Study Group	Statistical Parameters					*p*-Value *
		Mean	SD	Median	Q_1_–Q_3_	Min.–Max.	
Albumins (mg/mL)	CD	26.42	25.25	15.75	7.90–46.95	0.44–109.50	<0.0001 **
	UC	38.96	34.07	32.70	12.90–53.40	0.80–163.30	
	Control	78.40	87.74	43.20	12.10–101.90	8.90–266.70	
Transferrin (mg/mL)	CD	3.21	3.75	1.32	8.85–5.00	0.15–14.01	=0.3548
	UC	2.70	2.91	1.55	0.94–3.34	0.18–10.85	
	Control	4.22	3.57	1.90	1.60–8.34	0.46–0.85	
Transthyretin (ng/mL)	CD	3.14	2.48	3.02	2.51–3.34	0.52–19.20	=0.0001 **
	UC	2.83	1.22	2.82	2.56–3.11	0.27–8.20	
	Control	1.32	0.56	1.40	0.94–1.66	0.37–2.24	

* Statistical significance for the model used. All between-group comparisons were controlled for the patients’ age and gender. ** Post hoc multiple comparisons. Albumins: CD vs. UC, *p* = 0.0414; UC vs. controls, *p* = 0.0001; CD vs. controls, *p* = 0.0060. Transthyretin: CD vs. UC, *p* = 0.7745; UC vs. controls, *p* = 0.0048; CD vs. controls, *p* = 0.0003.

**Table 4:** Concentrations of albumins, transferrin and transthyretin according to the basic clinical characteristics of the patients with IBD.

	Albumins (mg/mL) Me [Q1–Q3]	Transferrin (mg/mL) Me [Q1–Q3]	Transthyretin (ng/mL) Me [Q1–Q3]
Disease duration			
<5	19.7 [13.4–42.0]	1.3 [0.9–2.9]	2.8 [2.3–3.1]
5–10	12.6 [7.2–47.3]	1.9 [1.0–5.2]	3.0 [2.5–4.3]
>10	27.8 [12.8–51.5]	1.3 [0.7–3.3]	3.0 [2.6–3.3]
*p*-value	0.4217	0.4213	0.2840
Biology therapy			
YES	19.4 [10.3–46.7]	1.3 [0.9–4.2]	3.1 [2.6–3.3]
NO	37.2 [13.8–63.9]	1.5 [0.2–2.9]	2.1 [1.9–3.1]
*p*-value	0.0753	0.3086	0.01224 *
Surgery history			
YES	31.1 [12.1–47.2]	2.1 [1.2–6.7]	3.0 [2.5–3.3]
NO	17.6 [9.6–47.6]	1.3 [0.8–3.1]	2.9 [2.4–3.2]
*p*-value	0.5300	0.1164	0.6717

* Univariate analyses. Post hoc multiple comparisons. Albumins: remission vs. moderate, *p* = 0.0252; moderate vs. severe, NS; remission vs. severe, *p* = 0.0132. Transthyretin: remission vs. moderate, NS; moderate vs. severe, *p* = 0.1900; remission vs. severe, *p* = 0.0143.

**Table 5:** Concentrations of albumins, transferrin and transthyretin according to the UC activity.

	Albumins (mg/mL) Me [Q1–Q3]	Transferrin (mg/mL) Me [Q1–Q3]	Transthyretin (ng/mL) Me [Q1–Q3]
Partial Mayo Score			
0	42.5 [32.9–70.1]	1.4 [1.0–3.3]	3.1 [2.6–37.4]
2	28.5 [12.8–47.2]	2.0 [0.5–3.4]	2.7 [2.1–2.9]
3	17.6 [12.8–53.4]	2.2 [0.9–3.6]	2.7 [1.9–3.1]
*p*-value	0.0151 *	0.8247	0.2583
Montreal Classification			
E1	43.5 [26.3–67.6]	2.7 [1.5–7.0]	3.2 [2.9–5.7]
E2	23.9 [12.8–42.0]	1.4 [0.2–3.4]	2.8 [2.6–3.1]
E3	38.1 [16.8–70.1]	1.5 [1.1–2.9]	2.6 [1.9–3.1]
*p*-value	0.5090	0.6870	0.1897
			
S0	23.9 [7.8–47.2]	1.4 [1.1–3.3]	3.1 [2.0–3.2]
S1	39.4 [17.2–42.0]	1.8 [1.0–2.9]	2.8 [2.6–3.2]
S2	28.1 [12.8–73.1]	2.3 [0.9–3.6]	2.7 [2.1–2.9]
S3	58.5 [46.9–70.1]	1.2 [0.9–1.5]	2.4 [0.3–4.4]
*p*-value	0.6145	0.8998	0.4560

* Univariate analyses. Post hoc multiple comparisons. Albumins: Partial Mayo Score 1 vs. 2, *p* = 0.0271; 1 vs. 3, *p* = 0.0091; 2 vs. 3, *p* = 0.0332.

**Table 6:** Concentrations of albumins, transferrin and transthyretin according to the Montreal classification of CD activity.

	Albumins (mg/mL) Me [Q1–Q3]	Transferrin (mg/mL) Me [Q1–Q3]	Transthyretin (ng/mL) Me [Q1–Q3]
Age at onset (years)			
A1 <16	9.4 [4.0–36.2]	1.3 [1.0–5.3]	3.2 [2.0–3.5]
A2 17–40	17.2 [10.5–47.3]	1.3 [0.8–5.2]	3.0 [2.5–3.3]
A3 >40	23.6 [16.9–38.0]	2.8 [1.4–5.1]	2.5 [2.5–2.9]
*p*-value	0.0488 *	0.7563	0.4829
Localization			
L1 Ileum	13.4 [7.2–25.8]	1.2 [0.8–2.4]	2.9 [2.5–3.3]
L2 Colon	46.8 [11.1–52.9]	3.2 [0.3–6.2]	2.4 [2.3–3.3]
L3 Ileum + colon	21.6 [8.6–47.2]	1.7 [0.9–8.3]	3.1 [2.6–3.4]
*p*-value	0.2663	0.3985	0.4127
Course of the disease			
B1 No stenoses or fistulas	19.1 [11.1–32.4]	1.3 [0.8–6.2]	2.8 [2.3–3.4]
B2 Stenoses	15.3 [6.1–46.7]	1.5 [1.2–6.4]	3.1 [2.9–3.6]
B3 Fistulas	46.7 [6.1–52.9]	1.3 [0.9–5.2]	2.9 [2.4–3.1]
Perianal lesions	25.1 [1.3–50.6]	0.5 [0.3–2.1]	3.1 [2.7–3.3]
*p*-value	0.7083	0.7711	0.7006
CDAI			
<150	47.2 [14.3–58.8]	1.2 [0.5–1.9]	3.2 [2.6–3.4]
150–220	19.7 [11.1–46.7]	2.0 [0.9–6.4]	2.9 [2.5–3.3]
221–450	17.2 [6.1–52.9]	2.5 [1.3–5.2]	2.8 [2.4–3.3]
>450	13.7 [2.2–27.8]	0.9 [0.2–1.3]	2.7 [2.1–3.1]
*p*-value	0.0334 *	0.1008	0.4962

* A univariate analysis. Post hoc multiple comparisons. Albumins: A1 vs. A2, *p* = 0.0321; A1 vs. A3, *p* = 0.0124; A2 vs. A3, *p* = 0.0452; CDAI < 150 vs. CDAI 150–220, *p* = 0.0373; CDAI < 150 vs. CDAI 221–450, *p* = 0.0131; CDAI < 150 vs. CDAI > 450, *p* = 0.0014; CDAI 150–220 vs. CDAI 221–450, *p* = 0.7772; CDAI 150–220 vs. CDAI > 450, *p* = 0.5332; CDAI 221–450 vs. CDAI > 450, *p* = 0.5337.

**Table 7:** Concentrations of albumins, transferrin and transthyretin according to the nutritional status of the patients with IBD.

	Albumins (mg/mL) Me [Q1–Q3]	Transferrin (mg/mL) Me [Q1–Q3]	Transthyretin (ng/mL) Me [Q1–Q3]
BMI (kg/m^2^)			
<18.5	28.1 [6.1–5.6]	1.3 [0.9–2.0]	2.8 [2.1–3.0]
18.5–24.9	34.3 [12.3–51.5]	1.8 [0.9–4.8]	2.9 [2.5–3.2]
>25	18.4 [8.6–39.4]	1.2 [0.8–3.6]	3.1 [2.6–3.5]
>30	14.8 [9.6–29.1]	2.4 [1.2–5.2]	2.6 [1.8–3.1]
*p*-value	0.0251 *	0.6566	0.2153
Waist circumference (cm)			
Normal	27.8 [10.5–51.5]	1.3 [0.9–3.4]	3.0 [2.5–3.3]
High	16.8 [10.7–29.8]	1.4 [0.9–4.2]	2.7 [2.5–3.2]
*p*-value	0.0213 *	0.6057	0.2923
Weight reduction in 6 months (%)			
5–10	27.1 [6.6–51.5]	1.4 [0.8–3.7]	2.8 [2.5–3.2]
>10	20.6 [12.2–43.6]	1.5 [0.9–4.8]	3.0 [2.6–3.3]
*p*-value	0.0463 *	0.4265	0.4540
Adipose tissue (%)			
Low	22.6 [11.1–46.7]	1.3 [0.2–2.4]	2.6 [2.1–3.0]
Normal	34.1 [6.1–58.8]	1.4 [0.7–5.2]	3.1 [2.6–3.3]
High	13.7 [10.5–45.3]	1.6 [1.0–4.2]	2.9 [2.8–3.3]
*p*-value	0.04866 *	0.4542	0.1560

* Univariate analyses. Post hoc multiple comparisons. Albumins: BMI < 18.5 vs. 18.5–24.9, *p* = 0.0021; <18.5 vs. >25, *p* = 0.3741; <18.5 vs. >30, *p* = 0.4481; 18.5–24.9 vs. >25, *p* = 0.0127, 18.5–24.9 vs. >30, *p* = 0.0134; >25 vs. >30, *p* = 0.3281. Albumins: adipose tissue low vs. normal, *p* = 0.0423; low vs. high, *p* = 0.0731; normal vs. high, *p* = 0.0227.

## Data Availability

Not applicable.
